# Trajectories in Body Height, Body Weight, BMI, and Nutrition Status From 1979 to 1987: A Measurement-Based Analysis of 15,717 Male Adolescents From the Capital City of Montenegro

**DOI:** 10.3389/fpubh.2020.610358

**Published:** 2020-11-06

**Authors:** Stevo Popovic, Bojan Masanovic, Srdja Martinovic, Dusko Bjelica, Jovan Gardasevic

**Affiliations:** ^1^Faculty for Sport and Physical Education, University of Montenegro, Niksic, Montenegro; ^2^Montenegrin Sports Academy, Podgorica, Montenegro; ^3^Faculty of Law, University of Montenegro, Podgorica, Montenegro; ^4^Ministry of Defence, Government of Montenegro, Podgorica, Montenegro

**Keywords:** anthropometry, secular trend, body proportions, youngsters, Montenegro

## Abstract

**Introduction and Objective:** This study aimed to consolidate body height and body weight, as well as the body mass index and nutrition status data of the entire male population of adolescents from Podgorica, the capital of Montenegro, in order to estimate trajectories in ahead mentioned variables from 1979 to 1987.

**Methods:** The sample includes 15,717 male adolescents divided into nine groups according to their year of birth. The sample of variables includes body height, body weight, and body mass index, as well as nutrition status, which was presented based on BMI standardized categories (underweight, normal weight, pre-obese, obese). The descriptive statistics were expressed as a mean and standard deviation for each variable, while *post-hoc* test with ANOVA were employed to explore differences between the means.

**Results and Discussion:** This study did not find significant differences in the body proportions of the measured group of subjects during the observed period, but some descriptive differences were observed that might be of interest for further analysis, especially when it comes to nutritional status.

**Conclusions:** The novelty and the original contribution of this study is reflected in the fact that descriptive data from the second half of the 20th century have been published, which can significantly help to follow the secular trend of one of the tallest nations in the world from the beginning of the 20th century—Montenegro—which has specific body proportions.

## Introduction

Trends in men's body height have been monitored and analyzed around the world for a very long time, almost 250 years (1, 2), and have been attracting a great deal of attention from people all over the world. In recent decades, in most industrialized countries, gradual progressive changes in mean body height values can be observed (1). Average adult body height has dramatically increased in Montenegro too (3, 4). The cause of this might be much better lifestyles adopted by the population, and it might be the result of better living conditions and improved nutritional, hygienic, economic, and health status (5–7). However, there is a lack of data within this population, which will be further elaborated. Namely, it is necessary to mention that Montenegro is located on the slopes of the Dinaric Alps, an area that has historically been recognized as a habitat of above-average tall people [cited in (4)]. The unusual height of Montenegrins was first observed by Robert Ehrich, measuring the male inhabitants of this area in the early 20th century (8, 9). The following studies that estimate the average body height of Montenegrins appear at the beginning of the 21st century, and all of them confirm that Montenegrins are one of the tallest nations in the world (10–14). Among the above-mentioned studies, a lack of record-keeping is noticeable. There is a multi-decade gap of quality data, which made it impossible to conduct a precise analysis of the factors that influenced the mentioned trends. This type of data is available throughout Europe and the rest of the world, which allows a detailed analysis of the secular trends. A great example can be found in the Netherlands where the tallest population nowadays live. Dutch central government created a system of key registers based on digital data, collected by government organizations for administrative purposes and databases are available to scientists on the basis of which they claim with certainty that their people have become taller and heavier since 1981 and accurately determine the secular trend. However, the ahead mentioned gap in Montenegro will be partly alleviated by this study and briefly explained and compared to other nation in the discussion section.

Trends in men's body weight have also shown a tendency to increase in previous studies (15–19), which is a logical consequence because the increase in size and magnitude must be accompanied by an increase in body weight but the accumulation of excess adipose tissue is the cause as well. However, body weight is not a variable that was analyzed by Montenegrin scientists very much. Although the body weight and body mass indexes, as well as nutritional status, are very important variables in many settings, organized national surveys were not conducted in Montenegro. Each set of data that can be accessed within the Montenegrin population from the 20th century is beneficial for further public health investigations. Although several studies evaluate changes in body height and monitor underweight, overweight, or obesity problems in school children (not in adolescents), very few monitor average body weight and body mass index (BMI) changes for a longer period; such trend studies have only been emerging recently (20–22). However, they are limited to local samples and do not include a sufficient number of respondents to generalize the conclusions. For this reason, in anticipation of a new statistics act that would stipulate that reduce the administrative burden to a minimum and allow scientists to access government key registers for statistical purposes, the Ministry of Defense and the University of Montenegro started a joint project to digitalize all the available data from the old archives of the Yugoslav People's Army, mostly because each male adolescent had to pass a medical examination, and the results were stored in their medical records.

As one of the first outcomes of this project, this study brings together the body height, body weight, and body mass index data of an entire young male population of the capital of Montenegro, Podgorica, which is the largest city in the country, including almost 30% of the nation's population, to evaluate the possible trends from 1979 to 1987, for the purpose of collecting information on possible acceleration, as well as the trajectories (changes) of nutrition status in adolescence.

## Methods

The population of this retrospective cross-sectional study contains the entire male population of the capital city of Montenegro, Podgorica, measured during mandatory medical examinations to test their ability for military service. Most future recruits underwent this examination before they were 18 years old, but military service could be postponed until the age of 27, so some of the future recruits had their medical examinations later, which increased the average age in each generation. However, the detailed analyses in the results section described it precisely.

In the period from 20 April 1979 to 1 July 1987, 16,401 future recruits of the Yugoslav People's Army with permanent residence in Podgorica underwent this examination, but male adolescents that were born in 1957 (*n* = 108), 1958 (*n* = 115), 1959 (*n* = 156), and 1960 (*n* = 305) were excluded from the analysis because their numbers were not large enough to represent (and reliably describe) an entire generation. Consequently, the analyzed data in this study covers the sample of 15,717 future recruits (17.93 ± 64 years) divided into nine groups: 1,326 subjects that were born in 1961 (18.54 ± 95 years), 1,927 in 1962 (18.22 ± 49 years), 1,859 in 1963 (17.92 ± 69 years), 1,021 in 1964 (17.77 ± 77 years), 1,822 in 1965 (17.82 ± 7 years), 2,346 in 1966 (17.91 ± 62 years), 1,634 in 1967 (17.88 ± 5 years), 1,897 in 1968 (17.72 ± 31 years), and 1,885 in 1969 (17.71 ± 17 years).

Anthropometric measurement was implemented in medical infirmaries, and subjects accessed the procedure in their underwear. From the sample measures that were collected for this research, body height and body weight were isolated; the body mass index is calculated using them. For body height and body weight assessment, a medical scale with moving weights with a stadiometer was used. Anthropometrical measurement was implemented by respecting the basic rules and principles of the International Biological Program (IBP). The body mass index was calculated based on the protocol handbook for physical form assessment connected to health (23), while the nutrition status was presented based on BMI standardized categories (underweight, normal weight, pre-obese, and obese) (24).

The data obtained in the research were processed using SPSS 20.0 software (Chicago, IL, USA). The descriptive statistics were expressed as a mean and standard deviation for each variable, while *post-hoc* test with ANOVA were employed to explore differences between the means.

## Results

An analysis of the average body height, body mass, and body mass index of young male subjects is shown in Table 1.

**Table 1:** Descriptive and Comparative Data of Male Adolescents from the Capital City of Montenegro enrolled in the study.

	**Mean** **±** **SD**			
**Year of Birth (Number of Subjects)**	**Age (years)**	**Body Height (cm)**	**Body Weight (kg)**	**Body Mass Index (kg/m^2^)**
1961 (*N* = 1,326)	18.54 ± 0.95	177.12 ± 6.98	68.70 ± 8.93	21.86 ± 2.24
1962 (*N* = 1,927)	18.22 ± 0.49	177.71 ± 6.80	68.74 ± 8.82	21.73 ± 2.16
1963 (*N* = 1,859)	17.92 ± 0.69	177.74 ± 6.89	68.46 ± 9.50	21.64 ± 2.48
1964 (*N* = 1,021)	17.77 ± 0.77	176.64 ± 7.37	67.35 ± 9.16	21.57 ± 2.48
1965 (*N* = 1,822)	17.82 ± 0.70	176.57 ± 6.88	68.13 ± 9.51	21.82 ± 2.52
1966 (*N* = 2,346)	17.91 ± 0.62	177.46 ± 6.77	68.45 ± 9.11	21.71 ± 2.50
1967 (*N* = 1,634)	17.88 ± 0.50	177.27 ± 7.25	68.79 ± 9.04	21.87 ± 2.39
1968 (*N* = 1,897)	17.72 ± 0.31	176.73 ± 7.06	68.33 ± 9.29	21.86 ± 2.27
1969 (*N* = 1,885)	17.71 ± 0.17	177.12 ± 6.95	69.01 ± 9.64	21.97 ± 2.59
1961–1969 (*N* = 15,717)	17.93 ± 0.64	177.19 ± 6.98	68.49 ± 9.26	21.79 ± 2.45

*No statistically significant difference between study groups were found*.

The average body height of the overall sample of male subjects was 177.19 ± 6.98 cm. The tallest group were subjects that were born in 1963 (177.74 ± 6.89), while shorter ones were subjects that were born in 1965 (176.57 ± 6.88). The average body weight of the overall sample of male subjects was 68.49 ± 9.29 kg, while the heaviest subjects were those that were born in 1969 (69.01 ± 9.64), and the least heavy were those born in 1964 (67.35 ± 9.16). The average body mass index of the overall sample of male subjects was 21.79 ± 2.45 kg/m^2^, while the highest values were among subjects that were born in 1969 (21.97 ± 2.59 kg/m^2^), and the lowest values had those that were born in 1964 (21.57 ± 2.48 kg/m^2^). The *post-hoc* test with ANOVA were employed to explore differences between the means and no statistically significant difference between study groups were found.

From Table 2, it can be observed that, in the overall sample of subjects, 6% were underweight, 85.16% were normal weight, 8.11% were pre-obese, and 0.74% were obese. The highest percentage of underweight is in the group of subjects that were born in 1964 (8.74%), while the lowest percentage is in the group of subjects that were born in 1961 (3.77%). The highest percentage of subjects with normal body weight is in the group of subjects that were born in 1961 (88.99%), while the lowest percentage is in those that were born in 1968 (82.24%). The highest percentage of pre-obesity is in the group of subjects that were born in 1968 (10.33%), while the lowest percentage is in those that were born in 1963 (6.13%). Lastly, the highest percentage of obesity is in the group of subjects that were born in 1963 (1.08%), while the lowest percentage is in those that were born in 1962 (0.21%).

**Table 2:** The Nutrition Status of Male Adolescents from the Capital City of Montenegro enrolled in the study.

**Year of Birth**	**Total**	**Underweight**		**Normal weight**		**Pre-obese**		**Obese**	
	***N***	***N***	**%**	***N***	**%**	***N***	**%**	***N***	**%**
1961	(*n* = 1,326)	50	3.77	1,180	88.99	88	6.64	8	0.60
1962	(*n* = 1,927)	94	4.88	1,705	88.48	124	6.43	4	0.21
1963	(*n* = 1,859)	130	6.99	1,595	85.80	114	6.13	20	1.08
1964	(*n* = 1,021)	80	7.84	857	83.94	78	7.64	6	0.59
1965	(*n* = 1,822)	124	6.81	1,525	83.70	158	8.67	15	0.82
1966	(*n* = 2,346)	157	6.69	1,990	84.83	176	7.50	23	0.98
1967	(*n* = 1,634)	77	4.71	1,392	85.19	156	9.55	9	0.55
1968	(*n* = 1,897)	127	6.69	1,560	82.24	196	10.33	14	0.74
1969	(*n* = 1,885)	104	5.52	1,580	83.82	184	9.76	17	0.90
1961-1969	(*n* = 15,717)	943	6.00	13,384	85.16	1,274	8.11	116	0.74

## Discussion

This study aimed to make a specific contribution in an attempt to resolve the centuries-old dilemma regarding the specifics of the body composition of Montenegrins. It was known that Montenegrins were the tallest nation in the world at the beginning of the 20th century (3, 8, 10) and that this fact has changed little in the last 100 years; Montenegrins were the second tallest nation at the beginning of the 21st century (4), just behind the Dutch (25). However, it remains unknown whether the growth trend was continuous or whether certain periods particularly influenced the growth and development of Montenegrins, and there are no scientific studies that would aid in answering this research question.

A deeper analysis and knowledge of specific growth and development trends would significantly help, first, in explaining the specifics of body height and other indicators of body composition, as well as providing a significant context for the further assessment of growth and development of Montenegrins in the future. Nevertheless, the answer could not be determined that far, and it will not be after the publication of this study. However, this study will, to a certain measure, help to reduce the existing gap and initiate the need for studies with relevant data to emerge that scientists could rely on in the future.

As a large number of scientists believe that the secular trend has not concluded in this population, the significance of this issue is gaining in importance, not only among physical anthropologists, but also among researchers from other related fields, because body height, as well other parameters that describe human body composition, are very important in many areas of human life (26). For example, from the fact the impact of better material living standards make people taller, the body height became a relevant indirect measure for living conditions in the periods for which little or no other data is available.

The fact that Montenegrins have grown by 6.36 cm in a little over 100 years (3), and that it is not known within what dynamics this growth took place, any study that would fill the gap in the database is welcome. The best comparative example is the Netherlands where the tallest people currently live but also where the secular trend is over. Dutch have grown by 14.37 cm in a little over 100 years (2, 25), much more than Montenegrins. So, if hypotheses are right that the secular trend in Montenegro is not over, there is a real possibility that Montenegrins will once again become the tallest nation in the world as soon as they reach the maximum body height.

One of the most important steps in proving the above claim is the capital city, which is partially, perhaps the most important part for analysis, since 30% of the entire Montenegrin population lives in it today, while three other Montenegrin cities have been processed too, namely Cetinje, Niksic, and Bar (20–22). The results of the research in the first two mentioned cities, which together with Podgorica, make up the central region of Montenegro, can already be very interesting in interpreting the obtained results, while the results from other Montenegrin cities are expected shortly, since the work on the digitization of the entire national data remains in process. It is interesting to note that particular attention will be paid to the results that will be obtained in research on the body composition of future recruits in cities across the northern and southern regions (at present only Bar is available), because it is generally known that there are traditional socio-economic and demographic differences between people in these regions (27).

Nevertheless, it is necessary to return to the discussion of the results of this study, in which the entire population of the capital of Montenegro is processed. In the 1980s, Podgorica was, demographically speaking, a completely different city. At that time, there were 132,290 inhabitants in Podgorica (22.64% of the total population of Montenegro), while today's Podgorica officially has 185,937 inhabitants, which represents 29.99% of the total population (28, 29). Changes in the number of inhabitants are conditioned by the constant development of the city in recent decades, and migrations to Podgorica were directed, primarily from rural areas of Montenegro, but also from smaller cities, as Podgorica offered many more opportunities to achieve a better lifestyle, both adult and younger population, as a secondary school and university center. It is worth noting that the increase of more than 50,000 inhabitants represents a significant figure for a small population such as Montenegro, while there is a suspicion that this number is even higher, as there are many citizens of the capital whose official residences are still in their home towns, since they are not significantly geographically dislocated.

The population of today's capital could, methodologically speaking, almost be a representative sample of the entire Montenegrin population, given that Podgorica is inhabited by people from all parts of Montenegro. However, the study in which Popovic (3) processed body height, primarily the entire population (183.36 cm), then the region (north = 183.01 cm; middle = 183.58 cm; south = 182.55 cm), but also Montenegrin cities (Podgorica = 182.04 cm), clearly indicates that huge gap reached over several decades do not allow the data from Podgorica to be used as a representative sample of the Montenegrin population when it comes to body height. Also, the results from Popovic's study (3) cannot even be compared with the data collected from the capital city in the 1980s, due to the large migrations from rural areas to Podgorica over last few decades. However, it is interesting to discuss the fact that the average body height of Montenegrins from the beginning of the 20th century was 177 cm, while a similar average was determined in the 80s in Podgorica (177.19 cm) that is only 0.19 cm higher. In contrast, current inhabitants of Podgorica are 182.04 cm tall, which is about 5 cm more, and the difference was achieved in a much shorter period. For that reason, the research question again arises as to what influenced such a drastic change: migration, socio-economic factors, or something completely different? The results of some local studies from Niksic, where the citizens of this city were on average 178.58 cm tall in the 1980s (20), or 184.57 cm at the beginning of the 21st century (3), can aid in answering this question, Cetinje, where the citizens of this city, on average, were 178.38 cm tall in the 1980s (21), or 181.25 cm at the beginning of the 21st century (3) and Bar, where the citizens of this city were on average 175.82 cm tall in the 1980s (22), and 182.13 cm at the beginning of the 21st century in Bar and Ulcinj (3). On the hand, Dutch have grown by 6.50 cm in since 1981 (2, 25), similarly like Montenegrins in this period of time, and make this research question more complex. However, altogether it leads us to collect a vast amount of data that would reveal the true picture of the growth and development trend of the Montenegrin young male population during the 20th century. Therefore, it is necessary to wait for the results of other ongoing studies in other Montenegrin cities and municipalities and attempt to access data from other periods; it will then be possible to answer much more precisely the research question of this study and related questions regarding the growth trends of the young male Montenegrins.

In contrast, with body weight, body mass index, and nutritional status, the situation is much more complex with regards to previous studies. There are no national studies from the beginning of the 20th century, as is the case with body height, but also in the later period. Studies can be found that are reflected in student papers and research projects done on small samples. In accordance with all the above, the project of the Ministry of Defense and the Faculty of Sports and Physical Education of the University of Montenegro is a crucial activity, primarily introducing us to the body weight, body mass index, and nutritional status of Montenegrins at the national level. This study finds that the average body weight of the citizens of Podgorica in the specified period was 68.49 ± 9.26 kg, while the body mass index was 21.79 ± 2.45 kg/m^2^. However, as with body height, the same data have already been published from Niksic (Body Weight = 68.56 ± 8.66; BMI = 21.48 ± 2.27), Cetinje (Body Weight = 70.16 ± 9.17; BMI = 22.02 ± 2.58), and Bar (Body Weight = 68.33 ± 9.73; BMI = 22.06 ± 2.58), which significantly opens the way to answer research questions regarding body weight and body mass index, and is still not close to that answer (20–22). However, it is unfortunate that there are no studies that describe Montenegrins as is the case with the Dutch, which can be said that for each additional centimeter in body length, the average Dutchman has become 2.3 kg heavier between 1981 and 2018 (30), but there is hope that in Montenegro, too, all available data will become digitized and available very soon. From the local perspective, it is interesting to note that the study by Gardasevic et al. (31), although on a small sample, indicates the possibility of a certain positive trend when it comes to body weight, since they found obvious differences in 18-year-old boys from Niksic who weighed an average of 78.5 kg and their average body mass index was 22.9 kg/m^2^, much more in body weight than from the current study, while the body mass index remains in equal values.

Finally, it is worth noting that a large number of studies have examined the nutritional status of the school population in Montenegro, but also amateur and professional athletes, as well as adults; however, the problem that occurs is the same as with the previous variables, primarily because the results obtained cannot be adequately compared for several reasons. In the first place, no data depict the situation before the results obtained in this study (Underweight = 6.00%; Normal weight = 85.16; Pre-obese = 8.11; Obese = 0.74), while methodological principles question the comparison of the results from the 1980s and the results obtained at the beginning of the 21st century, in the same way as described in the part that was dedicated to body height. However, it is necessary to conclude that the nutritional status of the 1980s was at an enviable level and it points out to the fact that adolescents were much more physically active at the time, all the way to the peak reached in the mid-90s (32) and the availability of information technology on a larger scale. Insufficient activity among adolescents is a major concern from the mid-90s up to nowadays, and this kind of studies are more than welcomed.

It is also worth noting that this study has achieved its goal: specifically, it has notably supplemented the existing database regarding the body height, body weight, body mass index, and nutritional status of the Montenegrin male adolescents. A complete sample of the population of the capital gives us a clear picture of what the situation was in Podgorica on this issue in the 1980s. However, although the sample covered the entire male population and filled and existing gap, this study has limitations. The main limitation of this study was to consider the age category of the subjects as the population consists of subject who are younger than 18 years old on average, mostly due to the reason there are assumptions that growth and development does not end at age 18, as it is previously believed, but rather growth and development systematically continues in the torso after 18 years of age (Starc, personal communication), which would ultimately cause an increase in body height. On the other hand, the fundamental methodological limitations do not allow us to compare the existing results from different periods of the 20th and 21st centuries in which they exist in the first place, but also the fact that this study did not include the female population, which is equally interesting for the professional and scientific public. Therefore, the recommendations for future research refer to the further expansion of the existing database, both in the periods where there are individual research studies in certain geographical locations and in the periods when there are none. Although the conclusion of the mentioned project of the Ministry of Defense and the Faculty of Sports and Physical Education of the University of Montenegro will significantly enrich the existing database, further research must treat the female population much more carefully in order to make these results available.

## Data Availability Statement

The raw data supporting the conclusions of this article will be made available by the authors, without undue reservation.

## Ethics Statement

The studies involving human participants were reviewed and approved by Ministry of Defense. Written informed consent from the participants' legal guardian/next of kin was not required to participate in this study in accordance with the national legislation and the institutional requirements.

## Author Contributions

SP wrote the manuscript, performed analyses, and revised manuscript. BM wrote the manuscript, performed analyses, and revised manuscript. SM collected the data. DB overviewed previous studies and discussed the results. JG discussed the results. All authors contributed to the article and approved the submitted version.

## Conflict of Interest

The authors declare that the research was conducted in the absence of any commercial or financial relationships that could be construed as a potential conflict of interest.
